# Medical Nutrition Therapy (MNT) Evidence Update: Comparative Effectiveness of Dietary Programs for Reducing Mortality and Cardiovascular Events in Adults with Increased Cardiovascular Disease Risk

**DOI:** 10.1016/j.advnut.2025.100399

**Published:** 2025-02-26

**Authors:** Zahra Esmaeilinezhad, Gabriel Torbahn, Bradley C Johnston

**Affiliations:** 1 Department of Nutrition, College of Agriculture and Life Sciences, Texas A&M University, College Station, TX, United States; 2 Department of Pediatrics, Paracelsus Medical University, Klinikum Nürnberg, Nuremberg, Germany; 3 Department of Pediatrics, Obesity Research Unit, Paracelsus Medical University, Salzburg, Austria; 4 Department of Epidemiology and Biostatistics, School of Public Health, Texas A&M University College Station, TX, United States

**Keywords:** Mediterranean diet, low-fat diet, mortality, stroke, myocardial infarction

## Abstract

**Health services question:**

In adults with established cardiovascular disease (CVD) risk factors, as compared with minimal intervention, what is the most effective dietary program intervention, with or without pharmacological management, physical activity, and behavioral support cointerventions, for reducing risk of early mortality and major cardiovascular events based on the best available systematic review and network meta-analyses of randomized clinical trials (RCTs)?

**Bottomline:**

Based on 40 RCTs evaluating 7 dietary programs, moderate certainty evidence suggests that Mediterranean dietary programs (for example, high in vegetables, fruits, extra virgin olive oil, nuts, legumes, and fish), accompanied by varying cointerventions including pharmacological management (for example, statins), physical activity and behavioral support (for example, nutrition education, smoking cessation, and stress management), were superior to minimal interventions for reducing risk of all cause [1.7% absolute risk reduction (ARR)], cardiovascular mortality (1.3% ARR), stroke (0.7% ARR), and myocardial infarction (1.7% ARR) in patients with established CVD risk factors (for example, obesity, hypertension, dyslipidemia, or a previous cardiovascular event) over a 5-y period. Results from randomized trials with food provisions (for example, extra virgin olive oil, mixed nuts, primarily walnuts) among those living in Mediterranean regions had the largest treatment effects. Similarly, moderate certainty evidence demonstrated that low-fat dietary programs (for example, 20–30% total fat, <10% saturated fat, and high in fish, vegetables, and fruits together with varying cointerventions) were superior to minimal intervention for reducing all-cause mortality (0.9% ARR) and myocardial infarction (0.7% ARR) based on trials conducted in Mediterranean, North American, and Northern European regions. Network metaregression did not detect statistically significant differences in estimates when controlling for the presence of pharmacological management, physical activity, and behavioral support.

## Graphical abstract

**Figure:**
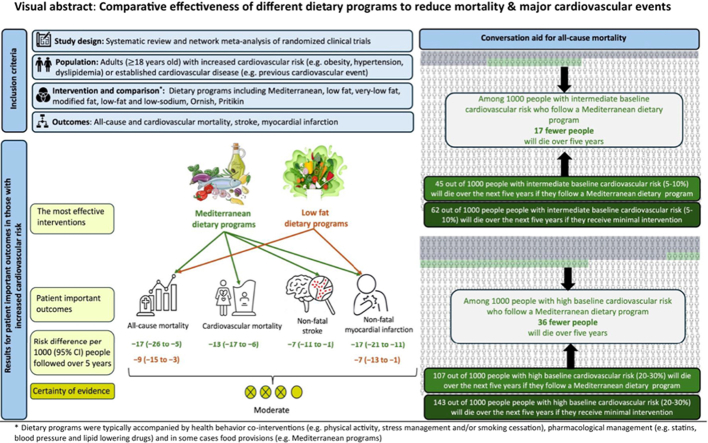

**Statement of significance**

This medical nutrition therapy (MNT) evidence update summarizes the findings from the highest-quality systematic review comparing the effectiveness of 7 dietary programs, with results presented as clinically intuitive absolute estimates of effect together with the certainty of these estimates based on Grading of Recommendations Assessment, Development and Evaluation (GRADE) methods for critically important outcomes to patients (for example, mortality). Designed for busy clinicians with limited time or appraisal skills, MNT evidence updates offers an accessible way to stay updated on the latest clinically relevant systematic reviews in context to clinical practice guidelines, evidence that will help guide health services decision making, and support personalized care based on outcomes and subgroup effects that matter most to patients and families, whereas also highlighting any major evidence gaps and needs for further research.

## Introduction

Among all modifiable behavioral risk factors that contribute to cardiovascular disease (CVD) risk at the population level, diet is thought to be the largest contributor [1,2]. Although there are various dietary programs assessed in randomized clinical trials (RCTs) for potentially reducing risk of early mortality and major cardiovascular events, clinicians often rely on single studies, whereas guideline recommendations often rely on surrogate outcomes (for example, lipid profiles) or evidence with low or very low certainty derived from observational studies [[3], [4], [5]]. We have summarized the best available systematic review evidence from RCTs assessing multiple dietary programs with or without pharmacological management, physical activity, or behavioral support on mortality and major cardiovascular events.

### Eligibility

We searched PubMed for high-quality systematic reviews with network meta-analyses (SRNMAs) of RCTs summarizing the evidence of different dietary programs in adults (≥18 y old) with increased CVD risk (for example, obesity, hypertension, and dyslipidemia) or established CVD (for example, previous cardiovascular event) published in the last 2 y (2023–2025). To optimize clinical utility, reviews had to report on mortality and major CVD outcomes (for example, stroke and myocardial infarction). High-quality SRNMA were determined using a modified, more stringent, Measurement Tool to Assess Systematic Reviews, version 2 (AMSTAR-2) appraisal criteria consisting of 18 questions [6]. To do so, we critically evaluated and ranked the quality (for example, comprehensiveness of search, screening, extraction, and risk-of-bias assessment) and interpretability (that is reporting absolute estimates of effect and GRADE certainty of the evidence) of reviews, with the interpretability questions representing the AMSTAR modification. The highest-quality SRNMA is summarized in the following section.

### Summary of findings

Of the 7 reviews selected for full-text screening, 2 potentially met our eligibility criteria (target population, intervention, comparator, and outcome), of which review by Karam et al. [7] was of the highest methodological quality based on modified AMSTAR-2 criteria (Supplemental Tables 1 and 2; Supplemental Text 1). Karam et al. (2023) included 40 RCTs (35,548 participants) reporting on 7 dietary programs for all-cause mortality and major CVD events (Table 1). The most effective programs compared with minimal interventions for reducing all-cause mortality were Mediterranean dietary programs (MDPs) and low-fat dietary programs. For study participants at intermediate risk of a cardiovascular event (5%–10%) over the next 5 y, a MDP probably results in 17 fewer [95% confidence interval (CI): −26, −5] all-cause cases of mortality per 1000 people followed. MDPs were also the most effective for the remaining cardiovascular outcomes, CVD mortality: 13 fewer, 95% CI: −17, −6; nonfatal stroke: 7 fewer, 95% CI: −11, −1, and nonfatal myocardial infarction (MI): 17 fewer, 95% CI: −21, −11 cases per 1000 people followed over 5 y. Low-fat dietary programs were also effective, with results showing 9 fewer (95% CI: −15, −3) all-cause cases of mortality, and 7 fewer (95% CI: −13, −1) cases of nonfatal MI per 1000 people over 5 y. For those at high CVD risk (20%–30% over the next 5 y), for those following an MDP, the absolute risk reductions were approximately double (Table 1).

**TABLE 1:** Summary of most effective dietary programs for mortality and cardiovascular events.

Outcomes	The most effective dietary program	No. studies (#participants)	Odds ratio (95% CI)^1^	Odds reduction (95% CI)	Risk difference per 1000 (95% CI) people followed over 5 y	Certainty of evidence
All-cause mortality	Mediterranean programs	10 RCTs (8075)	0.72 (0.56, 0.92)	−28% (−44%, −8%)	Intermediate baseline risk: −17 (−26, −5) High baseline risk: −36 (−58, −10)	Moderate
	Low-fat programs	16 RCTs (9243)	0.84 (0.74, 0.95)	−16% (−26%, −5%)	Intermediate baseline risk: −9 (−15, −3) High baseline risk: −20 (−33, −6)	Moderate
Cardiovascular mortality	Mediterranean programs	9 RCTs (8011)	0.55 (0.39, 0.78)	−45% (−61%, −22%)	Intermediate baseline risk: −13 (−17, −6) High baseline risk: −39 (−54, −19)	Moderate
Incidence of nonfatal stroke	Mediterranean programs	9 RCTs (7780)	0.65 (0.46, 0.93)	−35% (−54%, −7%)	Intermediate baseline risk: −7 (−11, −1) High baseline risk: −16 (−25, −3)	Moderate
Incidence of nonfatal MI	Mediterranean programs	9 RCTs (7895)	0.48 (0.36, 0.65)	−52% (−64%, −45%)	Intermediate baseline risk: −17 (−21, −11) High baseline risk: −42 (−53, −28)	Moderate
	Low-fat programs	12 RCTs (8105)	0.77 (0.61, 0.96)	−23% (−39%, −4%)	Intermediate baseline risk: −7 (−13, −1) High baseline risk: −18 (−31, −3)	Moderate

Abbreviations: CI, confidence interval; MI, myocardial infarction; RCT, randomized clinical trial.

1 Sensitivity analyses excluding trials with treatment arms including smoking cessation or drug treatment cointerventions revealed similar findings to our main analysis; however, loss of statistical significance was observed for low-fat dietary programs for all-cause mortality, nonfatal myocardial infarction, and unplanned cardiovascular interventions. Statistical significance was maintained for Mediterranean dietary programs outcomes, although they were based on the higher risk-of-bias trials.

All estimates for mortality and cardiovascular events were based on GRADE moderate certainty evidence. Network meta-analysis authors downgraded 1 level (from high to moderate) because of indirectness for the varying presence of cointerventions (in particular, pharmacological management, physical activity, and behavioral support) in trials comparing MDPs compared with minimal intervention. However, although covarying interventions could have been effect modifiers, network metaregression did not detect statistically significant differences in estimates when controlling for the presence of cointerventions (pharmacological management, physical activity, and behavioral support).

### Summary in context to other systematic reviews

On the basis of GRADE moderate certainty evidence, the best available SRNMA showed that MDPs are superior to minimal interventions for reducing all-cause and cardiovascular mortality, stroke, and MI risk [7]. The results from Karam et al. [7] align with a 2019 Cochrane systematic review with meta-analysis of RCTs on MPDs with respect to estimates and certainty of effects [8]. Although another systematic review with network meta-analysis of 17 RCTs comparing 4 diet programs has shown a superior protective effect of the MDPs on mortality and cardiovascular events, the absolute estimates were not reported, lowering the quality and interpretability of results for health service decision making [9]. With respect to observational studies, a 2020 systematic review with meta-analysis of cohort studies evaluating the effect of MDPs on primary prevention was the only study that assessed and reported certainty of evidence based on the GRADE approach. However, the review did not report absolute estimates of effect, again impeding the interpretability of results for decision-makers. Authors reported a relative risk reduction (RRR) in cardiovascular mortality of 21% (RR 0.79; 95% CI: 0.77, 0.82; 21 studies with 883,878 participants; low certainty of evidence), a RRR in fatal and nonfatal stroke by 20% (RR 0.80; 95% CI: 0.71, 0.90; 5 studies with 79,287 participants, moderate certainty evidence) and a RRR in fatal and nonfatal myocardial infarction by 27% (RR 0.73; 95% CI: 0.61, 0.88; 2 studies with 35,489 participants; moderate certainty evidence) [10].

### Summary of meaningful dietary components

Among the 12 RCTs on MPDs in Karam et al. [7] that reported mortality or major cardiovascular outcomes, there was considerable variability in study characteristics, including interventions (Supplemental Table 3). To better understand the application of the various dietary components, we relied on the largest MDP trial (PREDIMED (Prevención con Dieta Mediterránea): *n* = 7447 participants with ≥3 CVD risk factors; randomly assigned to 3 study arms and followed up over 4.8 y). PREDIMED included food provisions of extra virgin olive oil (≥ 4 tablespoons/d) in study arm 1, or provisions of mixed nuts (≥30 g/d) primarily walnuts in study arm 2, as compared with low-fat dietary advice in study (control) arm 3, while measuring dietary intakes using both biomarkers with evidence of validity (for example, urinary hydroxytyrosol, plasma α-linolenic acid) and memory-based recall methods (that is Mediterranean diet adherence screener) [11]. Authors reported that the intake of extra virgin olive oil was 7 compared with 27 g/d more in participants who were supplied mixed nuts and olive oil, respectively, and the intake of mixed nuts was 3 compared with 21 g/d more in participants who were supplied olive oil or mixed nuts, respectively, as compared with low-fat dietary advice group [11]. In addition to food provisions, study participants in the Mediterranean diet groups were suggested to consume daily vegetables (≥2 servings/d), fruits (≥3 servings/d), legumes (≥3 servings [450 g]/wk), and fish [≥3 servings (300–450 g)/wk], and were asked to replace red meat with white meat, although having an option to drink ≤1 glass of wine with a meal per day (≥7 glasses/wk) [11]. The intake assessments showed weekly increased intake of fish [0.3 serving (30–45 g)] and legumes [0.4 serving (60 g)] in both intervention groups as compared with the low-fat dietary advice group. However, changes in vegetables, fruits, red meat, and wine did not differ statistically between each of the 3 groups [11].

To interpret PREDIMED’s reported dietary intake data, the differential in interventions was primarily because of substitution (10% kcal) of refined olive oil with extra virgin olive oil intake, or as a result of increased nut intake (primarily walnuts at the expense of refined olive oil and carbohydrate food intake) alongside modest changes in other Mediterranean diet components (that is legumes and fish). The low-fat dietary advice group, by comparison, reported a modestly lower Mediterranean diet score based on the Mediterranean diet adherence screener, and importantly, an unsuccessful reduction in fat consumption (from 39% to 37%). Overall, the trial showed a reduction in major cardiovascular events when extra virgin olive oil or nuts were added to a Mediterranean-style diet as compared with a low-fat dietary advice control group that ultimately contained ∼37% fat among participants in Spain. It is reasonable to suggest that some individual dietary components, including extra virgin olive oil, mixed nuts (for example, walnuts), and legumes, with increased polyphenol intake in each of the Mediterranean diets, played a meaningful role [12,13].

### MDPs in context to cointerventions

In addition to differences in Mediterranean dietary patterns, other cointerventions (that is, pharmacological management, physical activity, and behavioral support including smoking cessation and stress management) have been considered across the 12 RCTs on MDPs reporting on mortality and/or major CVD events, as detailed in Supplemental Table 4. With respect to medication use, the most likely driver of effect modification, all 12 RCTs on MDPs allowed participants to continue any pharmacological management, or in some cases adjust medication use according to clinical practice guidelines, including antiplatelet drugs, β-blockers, lipid-lowering agents, angiotensin-converting enzyme inhibitors, calcium channel blockers, diuretics, anti-diabetic drugs, and hormone-replacement therapy. Pharmacological management was similar at baseline among all studies reporting medication as a baseline characteristic. The most commonly used medications after randomization were antiplatelet drugs such as aspirin (10 trials with intake ranging from 20% to 100%) and lipid-lowering agents such as statins (8 trials with intake ranging from 40% to 87%). With respect to other cointerventions, 4 out of 12 RCTs provided unequal advice on physical activity and behavioral support, including smoking cessation and stress management. Two of 12 studies conducted postrandomization adjustments for pharmacological therapy and physical activity, confirming the benefit of a Mediterranean-style diet for reducing cardiovascular events [11,14]. Moreover, metaregression analysis on 12 RCTs found no statistically significant difference when accounting for indirectness in cointerventions (in particular, pharmacological management, physical activity, and smoking cessation) in trials comparing MDP and minimal intervention [7].

### Adherence and effectiveness of MDPs in non-Mediterranean countries

Among the 12 RCTs included in Karam et al. [7] that reported mortality and major CVD events, 8 assessed dietary adherence. These studies used the Mediterranean diet adherence screener [11], blood and urine biomarkers [11,14,15], dietary records [[14], [15], [16], [17]], food frequency questionnaires [11,15], ad hoc questionnaires [18,19], and a 5-point Likert-like scale (which measured frequency of multiple Mediterranean food items) [20]. Because of the varied methods of assessing dietary adherence, different frequencies of follow-up (that is, multiple times compared with 1-time dietary adherence assessment) and different follow-up periods (for example, 1 to 5 y), inferences on diet pattern adherence are very challenging. Overall, the use of biomarkers for olive oil (urinary tyrosol and hydroxytyrosol) and nut consumption (alpha-linolenic acid) in the PREDIMED trial represent the most reliable adherence measures, at least for extra virgin olive oil and nut provisions, foods high in polyphenols with evidence of cardiometabolic benefits [12,13,21]. Otherwise, the studies tended to consistently show an increased intake of Mediterranean diet items in the intervention groups [11,[14], [15], [16], [17], [18], [19], [20]], and sustained adherence throughout the intervention period [11,[14], [15], [16], [17], [18], [19]]. Overall, although there is ongoing research to potentially identify valid biomarkers and metabolic signatures/profiles for dietary patterns [21], future trials need to better report on the quality and quantity of dietary intake using valid and reliable biomarkers and metabolic signatures at multiple time points to better determine how increased adherence to specific foods and dietary patterns may impact both surrogate (for example, lipids) and critically important outcomes to patients (for example, quality of life and mortality).

With respect to adherence to MDPs, an umbrella review of 13 meta-analyses of observational studies and 16 meta-analyses of RCTs provided evidence that greater adherence to the Mediterranean diet is associated with a lower risk of all-cause mortality, coronary artery disease, and myocardial infarction [22]. However, it must be noted that adherence may be higher in Mediterranean countries or that the provision of foods may ultimately be driving the reported efficacy of MDPs in RCTs. For example, results from RCTs of MDPs (for example, PREDIMED) in Mediterranean countries may not necessarily apply to non-Mediterranean countries because culturally related factors such as lifestyle (including social networks and walkability within cities), food habits, food preparation, and food availability can vary greatly between countries and regions [23]. For example, lifestyle factors (that is physical activity, sleep and napping, conviviality, limited television/screen time) in Mediterranean countries are likely to account for some of the reduced risks in mortality and cardiovascular events, as documented using the Medlife instrument in some observational studies [[24], [25], [26]]. Although we might conclude that MDPs are superior to low-fat programs or minimal intervention [7] based on RCTs, the efficacy of MDPs in North America and other countries has not been definitively established for mortality and major CVD events. For example, the PREDIMED trial demonstrated that a MDP (with provisions of either extra virgin oil or mixed nuts) reduces the absolute risk of cardiovascular mortality by 0.6% (6 fewer cases per 1000) and 0.4% (4 fewer cases per 1000) as compared with minimal control diet over ∼5 y of follow-up, a small but important difference [11,27]. Studies of similar scale and rigor (for example, dietary assessments that include valid biomarkers) with food provisions in non-Mediterranean countries do not exist, making direct comparisons and applicability of PREDIMED results outside of Mediterranean countries challenging. Notably, a small secondary prevention RCT (202 participants post-myocardial infarction) without food provisions, the lone MDP trial conducted in the United States to measure mortality risk found that the group assigned to an MDP as compared with the control group (usual care) followed for 3.8 y showed an absolute risk reduction of 0.029% (0.29 fewer cases per 1000) for cardiovascular mortality, a trivial and unimportant difference for individual patients [14]. For those living outside of Mediterranean countries, Table 2 summarizes the key foods, lifestyle factors, and barriers and strategies for implementing a Mediterranean diet and lifestyle [11,26,28].

**TABLE 2:** Applying Mediterranean diet and lifestyle interventions in non-Mediterranean countries.

Key foods in Mediterranean diet [28]	− Abundant use of polyphenol-rich extra virgin olive oil − High consumption of plant-based foods high in polyphenols (fresh vegetables, fruits, legumes, whole grains, and mixed nuts) − Moderate-to-high consumption of clean and sustainable sources of fish
Key foods in Mediterranean diet supported by the best evidence [11]	− Extra virgin olive oil (≥ 4 tablespoons/d) − Mixed nuts (primarily walnuts) (≥ 30 g/d) − Legumes [≥3 servings (450 g)/wk] − Fish [≥3 servings (300–450 g)/wk]
Key Mediterranean lifestyle health behaviors [26]	− Physical activity > 300 min/wk − Nap/Siesta ≤ 30 min/d − Hours of sleep = 6–8 h/d − Watching television/screen time ≤ 2 h/d − Conviviality/socializing with friends > 1 h/d − Collective/community sports ≥ 1 h/wk
Key Mediterranean lifestyle health behaviors supported by the best evidence [26]	− Physical activity > 300 min/wk − Nap/Siesta ≤ 30 min/d − Watching television/screen time ≤ 2 h/d
Barriers to address during nutrition counseling [28]	− Food accessibility − Food affordability − Ingrained habits − Unfamiliarity with culturally similar foods, recipes, and cooking methods
Strategies for applying Mediterranean dietary interventions [28]	− Personalized counseling to patients’ unique circumstance; set realistic goals − Replacing ultraprocessed snacks with healthier alternatives like mixed nuts and legumes and promoting clean fish as a simple and quick meal − Encouraging shared cooking to enhance adherence and enjoyment − Emphasize physical activities that fits into the patients’ lifestyle (for example, walking, household chores, dance, cycling, and community sports)

### Comparison of findings with current clinical practice guidelines

Clinical practice guidelines used by physicians and allied health professionals to treat those at increased CVD risk have made recommendations that align with our synopsis findings. Notably, based on GRADE methods, the Canadian Cardiovascular Society (2021) [29] recommended MDPs for managing dyslipidemia to prevent CVD risk (strong recommendation, high certainty evidence). The European Society of Cardiology (2021) [30] has recommended MDPs “or similar diets” for lowering the risk of CVD (class I recommendation, level A evidence), and similarly the American Heart Association and American College of Cardiology (2023) [31] have recommended MDPs (emphasizing vegetables, fruits, legumes, nuts, whole grains, and lean protein) in patients with chronic coronary disease to reduce the risk of CVD events (class I recommendation, level B evidence). Across all guidelines, MDPs represented the only intervention with strong or class 1 recommendations based on what is considered high certainty or level 1 evidence according to these organizations. By contrast, the best available SRNMA summarized here [7] has reported that MDPs have “moderate” certainty evidence for mortality and cardiovascular events, owing to issues of indirectness related to pharmacological management, physical activity, and behavioral support cointerventions. This means that there is considerable heterogeneity with respect to an MDP intervention, and we do not have high certainty for a specific, standardized MDP. On the basis of our findings in Supplemental Table 3, for those wishing to apply the study results on MDPs to patients for the potential reduction in mortality and major cardiovascular events, important additional issues of indirectness need to be considered. Additional issues that may further lower the certainty of evidence at a clinical implementation level include differences in the effectiveness of studies with and without food provisions and differences in studies conducted in Mediterranean compared with non-Mediterranean countries. Furthermore, assessments on the degree of behavioral and educational support by qualified personnel (for example, registered dietitians) compared with less qualified personnel (for example, physicians and nurses) would be helpful.

### Implications for clinical practice and future research

This high-quality comparative effectiveness review of a network meta-analysis of RCTs reports absolute estimates of effect as well as GRADE certainty of the evidence for mortality and major cardiovascular outcomes, summary evidence that can be shared using a conversation aid to support more fully informed clinical decisions, decisions that will likely be value and preference sensitive for each individual client or health service [32]. Our confidence in the estimates of effect applies primarily to those in Mediterranean regions, particularly when the Mediterranean diet and lifestyle are combined with food provisions (extra virgin olive oil, mixed nuts). With respect to future research, despite the cost, randomized trials of MPDs that provide food provisions and that measure mortality risk are needed in non-Mediterranean countries before strong recommendations can be justified globally.

## Author contributions

The authors’ responsibilities were as follows – BCJ: conceptualized the MNT evidence update framework; BCJ, ZE: screened studies independently, drafted the paper, and provided administrative support; ZE, GT: extracted data independently; GT: critically reviewed the paper for intellectual and clinical content; GT, ZE, BCJ: primary responsibility for final content; and all authors: read and approved the final manuscript.

## Funding

The authors reported no funding received for this study.

## Conflict of interest

BCJ is a GRADE working group member and a coauthor on the network meta-analysis summarized herein. BCJ has received a start-up grant from Texas A&M AgriLife Research to fund investigator-initiated research related to saturated and polyunsaturated fats. The grant was from Texas A&M AgriLife institutional funds from interest and investment earnings, not a sponsoring organization, industry, or company. BCJ also holds National Institute of Diabetes, Digestive and Kidney Diseases R25 funds to support training resources in evidence-based nutrition practice. The remaining authors declare that they have no known competing financial interests or personal relationships that could have appeared to influence the work reported in this paper.
