# Supplementary material for: Medical Nutrition Therapy (MNT) Evidence Update: Comparative Effectiveness of Dietary Programs for Reducing Mortality and Cardiovascular Events in Adults with Increased Cardiovascular Disease Risk
Source: Adv Nutr. 2025 Feb 26;16(4):100399. doi: 10.1016/j.advnut.2025.100399 (PMC11994918; doi:10.1016/j.advnut.2025.100399)
Supplement: Supplementary file 1 — Multimedia component 1 [file mmc1.docx]

**MNT Evidence Update: Comparative Effectiveness of Dietary Programs for Reducing Mortality and Cardiovascular Events in Adults with Increased Cardiovascular Risk**

Zahra Esmaeilinezhad et al.

**Supplementary Text 1. The search, screening, quality and interpretability assessment process**

We searched PubMed from January 1, 2023 to February 10, 2025. Our search identified a total of seven articles based on keywords related to diets (e.g., Mediterranean, low-fat, low-carbohydrate), major cardiovascular outcomes (e.g., mortality, stroke, myocardial infarction) and systematic review and network meta-analysis of randomized trials. After two authors independently screened these studies against our population, intervention, comparator and outcome criteria, two reviews were potentially eligible (Doundoulakis et al. (1); Karam et al. (2)). Subsequently, we independently assessed the methodological quality and interpretability of results using a modified version of AMSTAR-2, and Karam et al. (2023) was found to be the highest quality, most comprehensive and interpretable network meta-analysis (Supplementary Table 1 and 2).

**Supplementary Table 1. Reasons for exclusion of systematic reviews after full-text screening**

| **Author, year** | **Title** | **Reason for exclusion** |
| --- | --- | --- |
| Wallerer et al., 2024 (3) | Association between substituting macronutrients and all-cause mortality: a network meta-analysis of prospective observational studies | Study intervention (macronutrient substitution); Study design (network meta-analysis of observational studies) |
| Doundoulakis et al., 2024 (1) | Effects of dietary interventions on cardiovascular outcomes: a network meta-analysis | Methodological quality (low modified AMSTAR-2 [e.g. did not report absolute estimates; not comprehensive NMA with only 3 diet patterns and 17 trials included]) |
| Bonekamp et al., 2024 (4) | Diet in secondary prevention: the effect of dietary patterns on cardiovascular risk factors in patients with cardiovascular disease: a systematic review and network meta-analysis | Study outcomes (body weight, blood pressure, lipid profile) |
| Lesser, 2023 (5) | In adults at CV risk, Mediterranean-style or low-fat dietary programs vs. minimal interventions reduce all-cause mortality | Study design (commentary) |
| Li et al., 2023 (6) | Interventions for reducing blood pressure in prehypertension: A meta-analysis | Study outcome (blood pressure) |
| Bonekamp et al., 2023 (7) | Effect of dietary patterns on cardiovascular risk factors in people with type 2 diabetes. A systematic review and network meta-analysis | Study population (type 2 diabetes patients); Study outcomes (body weight, blood pressure, lipid profile, glycemia) |

**Supplementary Table 2. The quality and interpretability of included systematic reviews based on a modified version of AMSTAR-2 appraisal criteria***

| **Questions on methodological quality and applicability of results** | **Study:** Comparison of seven popular structured dietary programs and risk of mortality and major cardiovascular events in patients at increased cardiovascular risk: systematic review and network meta-analysis.  Karam G et al., *BMJ* 2023 Mar 29;380:e072003. |
| --- | --- |
| Item 1. **Did the research questions and inclusion criteria for the review include the components of PICO?** | Definitely yes  They explained PICOT items (page 2, lines 34 to 56, right column):   - Participants: adults (≥18 years) with two or more established risk factors for cardiovascular disease (e.g., hypertension, dyslipidemia, obesity, diabetes mellitus), or established cardiovascular disease (history of coronary artery disease, myocardial infarction, stroke or peripheral artery disease) - Intervention: popular named diet. - Comparison: minimal intervention, or with other cardiovascular risk reduction interventions (including other dietary programs) - Outcomes: all cause or cardiovascular mortality, or major cardiovascular events (stroke, non- fatal myocardial infarction, angina, heart failure, peripheral vascular events, atrial fibrillation, and unplanned cardiovascular interventions) - Time: at least nine months of intervention |
| Item 2 - Did the report of the review contain an explicit statement that the review methods were established prior to the conduct of the review and did the report justify any significant deviations from the protocol? | Definitely yes  The study protocol is registered with PROSPERO: CRD42016047939. (page 2, lines 14 to 18, right column). |
| Item 3. **Did the review authors explain their selection of the study designs for inclusion in the review?** | Definitely yes  They explained that they included any randomized controlled trials with eligible PICOT items. (page 2, line 34, right column). |
| Item 4 - Did the review authors use a comprehensive literature search strategy? | Definitely yes  Page 2, lines 18 to 32, right column:   - They searched at least three primary databases (5 in total): Embase, Medline, and CINAHL (Cumulative Index to Nursing and Allied Health Literature), AMED (Allied and Complementary Medicine Database) and Cochrane Central Register of Controlled Trials (CENTRAL) from inception to September 2021. - They searched ClinicalTrials.gov for unpublished and ongoing trials. - They searched reference lists from eligible trials and related reviews. - While they did not contact experts in the field, their search was comprehensive enough. - There was no language and time restrictions. - Search terms included extensive controlled vocabulary and keyword searches, and a very extensive search strategy for each primary database is provided in the Appendix. |
| Item 5. **Did the review authors perform study selection in duplicate?** | Definitely yes  The screening was performed by two independent reviewers. (page 3, lines 3 to 5, left column). |
| Item 6. **Did the review authors perform data extraction in duplicate?** | Definitely yes  Data extraction was performed by two independent reviewers. (page 3, lines 6 to 9, left column). |
| Item 7 - Did the review authors provide a list of excluded studies and justify the exclusions? | Definitely yes  They mentioned the number of excluded studies, the reason for exclusion and references in Figure 3 (PRISMA follow diagram, page 5) and Table S2 (pages 19 to 25). |
| Item 8. **Did the review authors describe the included studies in adequate detail?** | Definitely yes  They reported the full list of included studies characteristics in supplementary table (Table S1, pages 14 to 18). |
| Item 9 - Did the review authors use a satisfactory technique for assessing the risk of bias in individual studies that were included in the review? | Definitely yes  Reviewers assessed risk of bias independently and in duplicate by using the Cochrane risk of bias tool and resolved disagreement by discussion or consultation with a third reviewer (page 3, lines 9 to 18, left column).  They judged risk of bias for 7 domains and categorized studies as low risk of bias if three key domains (random sequence generation, allocation concealment, and missing participant outcome data) were all clearly reported to judge as low risk of bias: 1) random sequence generation, 2) allocation concealment, and 3) missing participant outcome data (Table S1, pages 14 to 18; Tables S72 to S77, pages 87 to 90). |
| Item 10. **Did the review authors report on the sources of funding for the studies included in the review?** | Definitely yes  They reported at the end of the article. (page 10, lines 64 to 67, left column and lines 1 to 9, right column). |
| Item 11. **If meta-analysis was performed did the review authors use appropriate methods for statistical combination of results?** | Definitely yes  They have stated explicitly in the PROSPERO review protocol the principles on which they based their decision to perform meta-analysis of data from the included studies.  They used Bayesian random effects models to obtain the pooled direct estimates and corresponding forest plots of the available direct comparisons. They assessed heterogeneity between randomized controlled trials for each direct comparison with visual inspection of the forest plots and the I^2^ statistic.  They considered two effect modifiers that were modelled as present or absent if they were included in an overall dietary program: exercise and behavioral support. They performed a network meta-regression assuming a common coefficient across comparisons to explore the effect of exercise and behavioral support for each outcome. Authors conducted three sensitivity analyses were conducted by restricting studies to trials with individuals who were overweight or obese, but who were otherwise healthy; those with a low risk of bias; and investigator initiated randomized trials, thus removing trials that were funded partly or wholly by diet companies.  For all outcomes, the analysis generated an odds ratio with a 95% confidence interval. Risk differences were calculated by applying the summary odds ratios to baseline risks (events per 1000 patients). |
| Item 12. **If meta-analysis was performed, did the review authors assess the potential impact of RoB in individual studies on the results of the meta-analysis or other evidence synthesis?** | Definitely yes  They assessed RoB for **individual studies. (Table S1, pages 14 to 18).** |
| Item 13 – Did the review authors account for risk of bias in primary studies when interpreting/ discussing the “overall” results of the review? | Definitely yes  Authors reported RoB based on the overall results indicating 27 were high RoB and 13 were low RoB. In addition, authors reported results based on GRADE certainty of evidence, which included assessing the domains of RoB. (Tables S73 to S77). |
| Item 14. **Did the review authors provide a satisfactory explanation for, and discussion of, any heterogeneity observed in the results of the review?** | Definitely yes  Authors assessed heterogeneity between randomized controlled trials for each direct comparison with visual inspection of the forest plots and the I^2^ statistic. For specifics, see answers to item 11 above.  Finally, they assessed heterogeneity (inconsistency) as part of the GRADE assessment of certainty evidence. (Tables S73 to S77). |
| Item 15 – If they performed quantitative synthesis did the review authors carry out an adequate investigation of publication bias and discuss its likely impact on the results of the review | Probably yes  They investigated publication bias as a domain of GRADE and reported it as not applicable in Tables S73 to S77. Authors did not provide funnel plot and results of statistical tests (Begg’s, Egger’s tests) given the limitations of trying to assess publication bias in network meta-analysis. Of note, the literature search was very comprehensive (5 databases and extensive search strategy) thus limiting the risk of publication bias. |
| Item 16. **Did the review authors report any potential sources of conflict of interest, including any funding they received for conducting the review?** | Definitely yes  The authors described their funding sources and how they managed potential conflicts of interest. (page 10, lines 1 to 9, right column). |
| Item 17. Did the review authors account for the overall certainty of evidence (eg, using GRADE or NutriGrade) for each of your outcomes with a meta-analysis when interpreting/discussing the results of the review? | Definitely yes  They rated the certainty of evidence for each network estimate using the GRADE framework, which classifies evidence as high, moderate, low, or very low certainty. (page 5, lines 27 to 30, left column and lines 1 to 30, right column; page 6, lines 1 to 6, left column; Tables S73 to S77). |
| Item 18. For dichotomous outcomes (e.g. yes or no variables such as mortality), did the review authors present the pooled meta- analytic data as an absolute estimate of affect (risk difference/absolute risk reduction, number needed to treat)? | Definitely yes  Outcome were dichotomous and they reported the absolute risk reduction (ARR) per 1000 patients followed over five years for all outcomes. |

Abbreviations: AMSTAR: A Measurement Tool to Assess Systematic Reviews, version 2; RoB: Risk of Bias.

* We added two questions (items 17 and 18) and we modified the response options from three (Yes, Partial yes, No) to four response options including (‘Definitely yes’, ‘Probably yes’, ‘Probably no’, and ‘Definitely no’). ‘Definitely yes’ indicates that the item is well reported and judged to be high quality.

**Supplementary Table 3. Characteristics of RCTs of Mediterranean-style diet programs for mortality and major cardiovascular outcomes**

| **Author year; country** | **Population; sample size; follow-up; outcomes** | **Intervention** | **Control** | **Method(s) of dietary intake assessment** | **Degree of adherence** |
| --- | --- | --- | --- | --- | --- |
|  |  | **Mediterranean dietary components** |  |  |  |
| **Mediterranean countries** | | | | | |
| de Lorgeril 1994; France (8) | Secondary prevention: Participants <70 years old with MI in the last 6 months  n=605  Follow-up: 5 years  Outcomes: All-cause mortality, cardiovascular mortality, non-cardiovascular mortality, non-fatal MI, angina, heart failure, pulmonary and peripheral embolism, venous thrombophlebitis, food intake | Components: “More bread, more root vegetables and green vegetables, more fish, less meat (beef, lamb, and pork to be replaced with poultry), no day without fruit, butter and cream to be replaced with margarine supplied by the study. Because the patients would not accept olive oil-traditional to the Mediterranean diet-as the only fat, a rapeseed (canola) oil-based margarine (Astra-Calve, Paris, France) was supplied free for the whole family to all experimental subjects. Moderate alcohol consumption in the form of wine was allowed at meals.”  Counseling: “Initial 1 hour session with cardiologist and dietician, and dietetic counselling at each follow-up.”  Food provision: “Rapeseed (canola) oil-based margarine high in linoleic acid supplied to participants. This margarine had a composition comparable to olive oil with 15% saturated fatty acids, 48% oleic acid but it includes 5.4% of 18:1 trans fatty acids. However, it was slightly higher in linoleic (16.4 vs 8.6%) and more so in alpha-linolenic acid (4.8 vs 0.6%), a fatty acid markedly higher (3-fold) in the plasma of the Cretan cohort in the Seven Country study compared to that of Zutphen (Netherlands). The oils recommended for salads and food preparation were rapeseed and olive oils exclusively.” | Minimal intervention:  “Control patients received no dietary advice apart from that of hospital dieticians or attending physicians.” | 24-hour recall, FFQ and biomarkers (plasma fatty acids) were measured at baseline and weeks 8, 52, 104. | “Eight weeks later, the experimental group had decreased their intake of saturated fat, cholesterol, and linoleic acid while increasing that of oleic and alpha- linolenic acid. After 52 weeks, there were higher concentrations of oleic, alpha-linolenic, and eicosapentaenoic acids and reduced concentrations of stearic, linoleic, and arachidonic acids in the experimental group. The increase in eicosapentaenoic acid was probably related to alpha linolenic acid since intake of fish was not significantly increased. In terms of foodstuffs the experimental group had a significantly higher intake of bread, fruit, and margarine; and a lower intake of butter, cream, meat, and delicatessen such as ham, sausage, and offal.” |
| Munoz 2007; Spain (9) | Secondary prevention: Participants between 30 to 79 years old, suffering from MI or angina in the last 6 years  n=983  Follow-up: 3 years  Outcomes: All-cause mortality, cardiovascular mortality, non-fatal MI, non-fatal-stroke, unstable angina, heart failure, arrhythmias, coronary artery revascularization, HRQoL cardiovascular risk factors (BP, blood lipids, glycemic indices, anthropometrics) | Components: Traditional Mediterranean diet “pulses, fruits, vegetables, fish, and olive oil, reduce total fat intake to 30% or less of total energy intake, achieve the reduction in saturated fats by replacing them in part with monounsaturated and polyunsaturated fats from both vegetable and marine sources, as well as complex carbs. Increase the intake of fresh fruits cereals and vegetables, reduce total calorie intake when weight reduction is needed, and reduce salt and alcohol intake use blood pressure is elevated.”  Counselling: “General practitioners (GPs) were reminded quarterly to provide patients in the intervention group with recommendations on healthy lifestyle, physical exercise (GPs offered various options, such as walking and cycling), hypo-caloric diet, and counselling to quit smoking if applicable. GPs also were asked to adjust life-saving treatments (i.e., angiotensin-converting enzyme inhibitors, aspirin, beta blockers and statins) to control risk factors according to the clinical practice guidelines, use the drugs recommended for secondary prevention if no contraindications, and request two laboratory tests each year.”  Food provision: None. | Usual care:  "Control group patients were only contacted on two occasions by telephone or mail to obtain clinical information, at the beginning and at the end of the study. Their GPs did not receive any letters reminding them to offer patients counselling and additional care." | Not reported. | Not reported. |
| Gianuzzi 2008;  Italy (10) | Secondary prevention: Participants $\leq$75 years old who had a recent MI (in last 3 months)  n=3,241  Follow-up: 3 years  Outcomes: All-cause mortality, cardiovascular mortality, non-fatal MI, non-fatal stroke, angina pectoris, heart failure, urgent unplanned revascularization procedure, major cardiovascular events (cardiovascular mortality plus nonfatal MI and nonfatal stroke), major cardiac events (cardiac death plus nonfatal MI), cardiovascular risk factors (BP, blood lipids, glycemic indices, anthropometrics), physical activity, stress and dietary habits | Components: “Healthy Mediterranean diet”. They cited a JAMA paper (11), however composition of the Mediterranean diet was not stated in the referenced publication.  Detection of a Mediterranean-like diet was based on a questionnaire with “questions focused on the frequency of usual consumption of cooked. vegetables, raw vegetables, fruits, fish, olive oil, and butter.”  Counselling: “Comprehensive cardiac rehabilitation sessions with one-to-one support were held monthly from month 1 to month 6, then every 6 months for years. Each session consisted of 30 minutes of aerobic exercise, plus lifestyle and risk factor counselling lasting at least 1 hour and reinforcement of preventive interventions lasting approximately 30 minutes. To improve adherence to lifestyle modification and help patients adopt a positive role in the care of their own health, a booklet explaining how to deal with exercise, diet, smoking cessation, and stress management was distributed. The mutual support of family members was encouraged in ad hoc meetings. Pharmacological treatments were positively recommended to all patients.”  Food provision: None. | Usual Care:  “A letter was sent to family physicians recommending secondary prevention goals. After the standard, post-MI CRP, patients in the usual care group reported to their reference center only to undergo the 6-month and then annual scheduled assessments. After each assessment, a copy of the results of laboratory and exercise tests was forwarded to the family physician who was responsible for any further medical decisions.” | Ad hoc questionnaire focused on the frequency of usual consumption of  cooked vegetables, raw vegetables, fruits, fish, olive oil, and butter (each scored 1 to 4) was measured at 6 months, 1 year, 2 years, 3 years. | “Dietary score increased by 18%  in the intervention group and 14.1%  in the usual care group after 6 months.” |
| Lapetra 2017; Spain (12) | Primary prevention: High risk hypertensive participants  n=180  Follow-up: 27.6 months ± 5 (SD)  Outcomes: Fatal and non-fatal stroke, atrial fibrillation, ischemic heart disease, heart failure | Components: “Mediterranean-style diet” (not further described).  Counseling: “For at least 2 years, dietary advice was given every 3 months.”  Food provision: None. | Low fat  Components: “Low-fat diet according to American Heart Association guidelines.”  Counseling: “For at least 2 years, dietary advice was given every 3 months.” | Not reported. | Not reported. |
| Marcos-Forniol 2018;  Spain (13) | Secondary prevention: Participants $\geq$70 years old diagnosed with acute coronary syndrome.  n=127  Follow-up: 3 years  Outcomes: All-cause mortality, HRQoL, cardiovascular risk factors (BP, LDL-C, HbA1C), diet adherence. | Components: Not described, but Mediterranean Diet Adherence Scale (MEDAS), (1 point each):  Olive oil ≥1 tbsp/day  Fruit ≥1 serving/day  Vegetables/salad ≥1 serving/day  Fruit ≥1 and vegetables ≥1 serving/day  Pulses ≥2 servings/week  Fish ≥3 servings/week  Red wine ≥ 1 glasses/day  Meat < 1 serving/day  (White bread <1 serving/day and rice <1 serving/week) *or* whole-grain bread >5 servings/week.  Counseling: “The multidisciplinary team (nurse, nutritionist and physician) gave intervention group patients recommendations to improve lifestyle (physical activity and exercise, diet, weight management and smoking cessation), adhere to a MedDiet and maintain physical function and changed the drug treatment if required to improve risk factor goals for CVD secondary prevention. Moreover, the intervention group patients attended physician appointments every three months (at the third, sixth and ninth months of the study) with a blood test performed the previous week. At these appointments, the physician assessed clinical, dietary, physical and psychosocial areas to make treatment modifications if necessary, and reminded patients of the initial physical activity, diet and other lifestyle recommendations. During the study period, both groups received their usual care at the discretion of their family physicians and cardiologists.”  Food provision: None. | Usual care:  “They received their usual care at the discretion of their family physicians and cardiologists.” | 136-item FFQ and an ad hoc questionnaire (based on 9 items of FFQ) were measured at baseline and after 12 months.  The dietary items included in the questionnaire were olive oil, fruits, vegetables, legumes, fish, wine, meat, white and whole bread.  (no information on validity was reported in their study, nor was there a referenced study.) | “A slight improvement in adherence to the Mediterranean diet was  observed in the intervention group at 12 months, but  had worsened in the control group, with a significant  difference. This improvement in the intervention group was due to a slightly better consumption of all dietary items, except oil and cereals, which did not change. Whereas there was a worsening in the control group, mainly due to a drop in the intake of vegetables and fish.” |
| PREDIMED 2018; Spain (14) | Primary prevention: Participants with type 2 diabetes or at least three cardiovascular risk factors (smoking, hypertension, elevated LDL-C, low HDL-C, overweight or obesity, or a family history of premature coronary heart disease)  n=7,447  Follow-up: 4.8 years  Outcomes: Composite of cardiovascular mortality, non-fatal MI, ischemic stroke, and individual coronary revascularization procedures, all-cause mortality, cardiovascular mortality, non-fatal MI, ischemic stroke | Components:  Extra-virgin olive oil ≥ 4 tbsp/day  Tree nuts and peanuts ≥3 servings/week  Fresh fruits ≥3 servings/day  Vegetables ≥2 servings/day  Fish (especially fatty fish), seafood ≥3 servings/week  Legumes ≥3 servings/week  Sofrito ≥2 servings/week  White meat instead of red meat  Wine with meals (optionally for habitual drinkers) ≥7 glasses/week  Discouraged: Soda drinks, commercial bakery goods, spread fats, red and processed meats.  Counseling: “Dietitians held individual and group dietary-training sessions at the baseline visit and quarterly thereafter.” Approximately 18 counselling sessions.”  Food provision: One liter of the extra-virgin olive oil per week per household for one intervention group and 30 g of mixed nuts per day per person (15 g of walnuts, 7.5 g of hazelnuts, and 7.5 g of almonds) for second intervention group. | Low fat:  Protocol: "Low fat diet according to AHA guidelines"  Trial registry: “Low-fat diet according to American Heart Association guidelines”  Defined for per protocol analysis purposes as <30% total fat  Components:  Low-fat dairy products ≥3 servings/week  Bread, potatoes, pasta, rice ≥3 servings/day  Fresh fruits ≥3 servings/day  Vegetables ≥2 servings/day  Lean fish and seafood ≥3 servings/week.  Discouraged: Vegetable oils, commercial bakery goods, nuts and fried snacks, red and processed fatty meats, visible fat in meats and soups, fatty fish, spread fats, sofrito.  Counseling: “Participants in the control group also received dietary training at the baseline visit and during the first 3 years of the study, they received a leaflet explaining the low-fat diet on a yearly basis. However, the realization that the more infrequent visit schedule and less intense support for the control group might be limitations of the study prompted us to amend the protocol in October 2006. Thereafter, participants who were assigned to the control diet received personalized advice and were invited to group sessions with the same frequency and intensity as those in the Mediterranean-diet group.” | 137-item FFQ validated in Spanish population and Mediterranean Diet Adherence Screener [MEDAS] instrument were measured annually.  Biomarkers (urinary hydroxytyrosol levels to confirm adherence in the group receiving extra-virgin olive oil, and plasma alpha-linolenic acid levels to confirm adherence in the group receiving mixed nuts) were measured at 1, 3 and 5 years. | “Adherence to the Mediterranean diet were  significantly higher in the two Mediterranean diet groups than in the control diet group  (p<0.0001 for all yearly comparisons from year 1 to 6 of follow-up) with the magnitude  of differences  ranging from 1.4 to 1.8 points.”  Percentage of positive response to Mediterranean diet score items ranged between 29.9% for wine (≥ 7 glasses/week) and 99.9% for olive oil as main culinary fat in the MeDiet+EVOO group, 32.3% for wine (≥ 7 glasses/week) and 97.5% for olive oil as main culinary fat in the MeDiet+Nuts group. And 25.1% for wine (≥ 7 glasses/week) and 97.1% for red or processed meats (<1 serving/day) in the control group. |
| **Non-Mediterranean countries** | | | | | |
| Singh 2002^*^; India (15)  (NB: NB: questions have been raised about the possibility of fabricated data; Karam et al., BMJ 2023 conducted sensitivity analysis with and without this RCT, showing similar pooled results) | Primary prevention: Participants ≥25 years old with hypercholesterolemia, hypertension, diabetes mellitus, or heart attack  n=1000  Follow-up: 2 years  Outcomes: All-cause mortality, non-fatal MI, fatal MI, sudden cardiac death, total cardiac endpoints, coronary angiography, angioplasty, coronary bypass surgery, food intake | Components: “Participants in both groups were advised to eat food substitutes that would provide a dietary intake similar to that recommended by the National Cholesterol Education Program (NCEP) in the step I prudent diet. This diet recommends that less than 30% of energy comes from total fat, less than 10% from saturated fat, and that less than 300 mg of cholesterol is consumed per day. They also recommended to consume at least 400 to 500 g of fruits, vegetables, and nuts per day, (i.e., 250 to 300 g of fruit, 125 to 150 g of vegetables, and 25 to 50 g of walnuts or almonds). This group was also encouraged to eat 400 to 500 g of whole grains, legumes, rice, maize, and wheat) daily, as well as mustard seed or soy bean oil, in three to four servings per day, which is consistent with recommendations from the Indian Consensus Group.”  Counseling: “All participants were asked to complete for one week a record of food intake, and to record occupational, household, and leisure time physical activity for weeks 1 to 4, then at weeks 4, 8, 12, and 24, then at 12 week intervals, to assess dietary adherence and exercise activity.  In both groups, completed diaries were checked by the dietician on every visit with the help of a questionnaire that used household measures and food models, to estimate caloric value of food portions and to reinforce dietary adherence. Patients with diabetes mellitus, angina pectoris, a history of myocardial infarction, or hypertension who visited the physician frequently, received more frequent dietary advice during the two years of follow up than those who did not. Patients from both groups were also advised to walk briskly for a minimum of 3–4 km, or to jog intermittently for a minimum of 10–15 minutes per day. All participants with CAD were instructed to take sublingual nitroglycerine and cease exercising in the event of chest pain, substantial sweating, weakness, or dyspnea. Smoking and alcohol consumption were discouraged, and we encouraged mental relaxation through yoga meditation techniques and breathing exercises in both groups. Appropriate drugs for angina pectoris, arrhythmias, raised blood pressure, diabetes, and other complications were provided to both groups.”  Food provision: None. | Low fat:  Components: “Participants in both groups were advised to eat food substitutes that would provide a dietary intake similar to that recommended by the National Cholesterol Education Program (NCEP) in the step I prudent diet. This diet recommends that less than 30% of energy comes from total fat, less than 10% from saturated fat, and that less than 300 mg of cholesterol is consumed per day.”  Counseling: “Control patients were given an information sheet on the step I prudent diet at each visit, intervention group patients were given a thorough explanation of the usefulness of the experimental diet, and the types of food that are rich in n-3 fatty acids. At all meetings, dieticians provided additional motivation to both groups to adhere to the advice about diet and exercise. Patients from both groups were also advised to walk briskly for a minimum of 3–4 km, or to jog intermittently for a minimum of 10–15 minutes per day. All participants with CAD were instructed to take sublingual nitroglycerine and cease exercising in the event of chest pain, substantial sweating, weakness, or dyspnea. Smoking and alcohol consumption were discouraged, and we encouraged mental relaxation through yoga meditation techniques and breathing exercises in both groups. Appropriate drugs for angina pectoris, arrhythmias, raised blood pressure, diabetes, and other complications were provided to both groups.” | 24-hour dietary record for Indian food composition-tables was measured at weeks 1 to 4,  then at weeks 4, 8, 12, and 24, then at 12 week intervals until the end of study (year two) | “Dietary intake of fruit,  vegetables, legumes, and oils increased in both groups but  much more so in the intervention group, suggesting that  participants adhered to the recommended diets.” |
| Søndergaard 2003; Denmark (16) | Secondary prevention: Patients aged 18 to 80 years with ischemic heart disease and total serum cholesterol level of ≥5.0 mmol/L (193 mg/dL)  n=131  Follow up: 1 year  Reported outcomes: All-cause mortality, total cholesterol, HDL, LDL, triglycerides, flow-mediated dilatation, nitroglycerin-mediated dilatation, endothelial-dependent response, food intake | Components: “at least 600 grams of fruits and vegetables daily, to modify the intake of fat, especially saturated fat from meat and dairy produce, to eat fatty fish at least once a week and preferably several times a week, to eat plenty of bread and cereals, and to replace refined, hard, animal margarine products with vegetable oils, preferably canola oil.”  Counseling: Dietary advice by a masters of science in clinical nutrition personnel and a specially trained research nurse. The first session was performed as a thorough interview lasting for at least 1 hour using the 24-hour recall method. Every patient had to describe the intake of foods and beverages for the past 24 hours, and the dietary advice was adjusted individually and repeated every third month.  Treatment with fluvastatin (40 mg every evening) was instituted for all patients.  Food provision: None. | Usual care  Components:  Booklets about heart-healthy diets that are usually delivered to patients in the coronary care unit (CCU). Single visit to dietitian (not participating in the study).  Treatment with fluvastatin (40 mg every evening) was instituted for all patients. | 24-hour recall at first session and 4-day diaries for control sessions (estimated portion sizes) | Not reported. |
| Tuttle 2008; USA (17) | Secondary prevention: Participants with post-MI (<6 weeks after first MI)  n=202  Follow up: 3.8 years  Outcomes: Individual and composite of all-cause mortality, cardiovascular mortality, non-fatal MI, non-fatal stroke, heart failure, unstable angina, individual cardiovascular risk factors (BP, lipid profile, BMI), food intake | Components: Total fat 30 to 40%  Sat fat $\leq$7%  Cholesterol <200 mg/day  Fresh fruits/vegetables $\geq$5 servings/day  Increased whole grains  Overweight/obese patients encouraged to lose weight  Consumption of cold-water fish 3 to 5 times/week  Omega-3 fatty acids >0.75%  MUFAs 20 to 25%  Oils from olive, canola, soybeans.  Counselling: “Two individual dietary counselling sessions from study dietitian in first month, with additional sessions at 3, 6, 12, 18, 24 months. There were also 6 groups sessions focused on behavioral modification and practical aspects of their assigned diets, including recipes, grocery shopping, and dining out. After completing 6 classes, participants were invited but not required to continue attending group sessions.”  Food provision: None. | Low fat:  Components:  Total fat <30%  Sat fat <7%  Cholesterol <200 mg/day  Fresh fruits/vegetables $\geq$5 servings/day  Increased whole grains  Overweight/obese patients encouraged to lose weight  Omega-3 fatty acids unchanged  Counseling: “Two individual dietary counselling sessions from study dietitian in first month, with additional sessions at 3, 6, 12, 18, 24 months. There were also 6 groups sessions focused on behavioral modification and practical aspects of their assigned diets, including recipes, grocery shopping, and dining out. After completing 6 classes, participants were invited but not required to continue attending group sessions.” | Self-reported 3-day food diaries were measured at 3, 6, 12, 18, and 24 months. | While they did not directly showed the degree of adherence, their dietary intake data showed increased intake of MUFA and omega 3 during 24 months in intervention group that could show their adherence to the Mediterranean diet. |
| Lehmann 2011; Germany (18) | Secondary prevention: CAD participants after coronary angiography, PTCA, or stationary treatment for CAD  n=105  follow-up: 3 years Outcomes: Cardiovascular mortality, non-cardiovascular mortality, non-fatal MI, severity and frequency of angina attacks, coronary bypass surgery, cerebral ischemia, PCI/sent, coronary artery calcium progression, stress and depression, cardiovascular risk factors (BP, lipid profile, BMI), lifestyle and dietary adherence | Components: Mediterranean-  type diet (not further described);  “Significant group differences in favor of the intervention were related to features of the Mediterranean diet, i.e. increased intakes of omega-3 fatty acids, fruits/vegetables, olive oil and a decreased intake of saturated fat.”  Counselling: “The program started with 3-day retreat then weekly 3-hour sessions for 10 weeks, then biweekly 2-hour sessions for rest of the 12 month intervention. Each session involved stress management and nutrition. Regular exercise and increased daily activity were strongly recommended. The stress management program was adopted. Patients practiced various relaxation techniques according to personal choice. Techniques taught included mindfulness meditation, guided imagery, yoga breathing techniques and body scan. Further elements included CBT (cognitive restructuring) and psychoeducational approaches (coping skills training).”  Food provision: None. | Minimal intervention:  Patients “received written information about stress reduction and diet by means of a booklet sent by mail after randomization.” | 5-point Likert scales (the exclusive use of olive oil/rapeseed oil, two portions of fatty fish/week, and five portions of fruits and/or vegetables/day) were measured at 3 years follow-up. | “Compliance with predefined nutritional recommendations was significantly higher in group who received Mediterranean dietary recommendations and they showed increased intakes of n-3 fatty acids, fruits/vegetables, olive oil and a decreased intake of saturated fat.” Mean (95%CI)  overall compliance  with nutritional  recommendations  72% (68–76) for intervention group and 62% (57–67) for control group. |
| Matz 2015; Austria (19) | Secondary prevention: Participants with ischemic stroke in past 3 months (also 78% had hypertension, 79% hyperlipidemia)  n=190  Follow-up: 2 years  Outcomes: All-cause mortality, severity of stroke,  cognitive subscale of the Alzheimer Disease Assessment Scale, occurrence of cognitive decline, functional outcome, vascular events, HRQoL, depression | Components: Changing from butter and other saturated fat to vegetable fat;  Consumption of rapeseed oil and vegetable margarines (≥60% fat) ≥20 g/day, calculated as oil;  Consumption of fatty fish at least 2 portions/week;  Consumption of fruit and veggies ≥500 g/day;  Choosing whole grain in all cereal products;  Choosing low-fat options in milk and meat products;  Choosing low-salt products;  Limiting sucrose intake;  In subjects who are not generally abstinent from alcohol, moderate consumption of alcoholic beverages with a limit of max. 2 units/day for men and 1 unit/day women;  Encouragement of consumption of dark chocolate, coffee, use of garlic, almonds, or hazelnuts.  Counseling: “Seven group dietary meetings of 60 min each and seven dietary individual meetings of 30 to 60 mins each recommended in study period (2 years). Study goals were defined as following: perform moderate or vigorous physical activity at least 3× to 5×/week, a body mass index <25 kg/m^2^ or weight loss and maintenance of at least 5% weight loss during the first year in obese individuals, individually defined dietary goals according to the composition of energy intake and the composition of foods, smoking cessation in patients who smoked, and a blood pressure <140/90 mm Hg (if diabetes mellitus, <130/85 mm Hg) of >75% of self-recorded measurements. Compliance with pharmacological treatment including lipid-lowering drugs, antithrombotic/oral anticoagulation therapy, and glucose-lowering drugs was targeted according to the treatment goals of stroke prevention European Stroke Organization (ESO) guideline.”  Food provision: None. | Usual care:  “The patients in the control group obtain stroke care according to standard guidelines and advice for secondary prevention during hospitalization. The patients get a full assessment at baseline, 12 months, and 24 months and receive regular phone calls to obtain adverse events (every eight-weeks during the first year and every 16 weeks during the second year).” | Not reported.  “Adherence to the study goals were checked by analyzing patient’s diaries, monthly calls from the coordinating center, and regular calls from the local study physicians.” | Not reported.  “Overall, the interventions were well accepted by patients  with stroke and adherence to interventional meetings was  good.” |
| Singh 2017; India  (20)  (NB: questions have been raised about the possibility of fabricated data; Karam et al., BMJ 2023 conducted sensitivity analysis with and without this RCT, showing similar pooled results) | Secondary prevention: Participants diagnosed with MI within the past 24 hours  n=406  Follow-up: 2 years  Outcomes: All-cause mortality, frequency of obesity, compliance with diet, weight loss, dietary adherence, food intake | Components: "In both diets, meat, eggs, hydrogenated oils, butter and Indian clarified butter were replaced with vegetarian substitutes, including cottage cheese, soya bean, and mustard oil [Intervention] patients were also advised to eat fruit, vegetables, pulses, nuts (almonds, walnuts; 50 to 100 g/day as yogurt or soup), and fish. The goal was to provide at least 400 g/day of fruits, vegetables, and nuts, and plenty of whole grains, including chickpeas, beans, and peas."  Counselling: “Dietary compliance was checked and reinforced daily during admission to hospital (10 to 30 days) by a dietitian and later one to 12 weekly, depending on the seriousness of the infarction and frequency of clinic visits.”  Food provision: None. | Low fat:  Components: "Control diet similar to National Cholesterol Education Program step 1 diet.  In both diets, meat, eggs, hydrogenated oils, butter and Indian clarified butter were replaced with vegetarian substitutes, including cottage cheese, soya bean, and mustard oil." | 24-hour dietary record was measured on days 3, 6, and 10 after infarction, then every week for six weeks and at 12, 24, 36 and 48 weeks for up to a year among survivors. | Significantly higher consumption of whole grains, fiber, nuts, fruits and vegetables, polyunsaturated fats, fish and soya bean and mustard oils, lower cholesterol and saturated fatty acids after one year was shown in Indo-Mediterranean diet.  Adherence to the diet and health recommendations were significantly higher in group Indo-Mediterranean diet than in control group. |

Abbreviations: BP: Blood Pressure; BMI: Body Mass Index; CAD: coronary Artery Disease; CBT: Cognitive Behavioral therapy; CRP: C-reactive Protein; GP: General Practitioner; HbA1C: Hemoglobin A1C; HDL-C: High Density Lipoprotein Cholesterol-C; LDL-C: Low Density Lipoprotein Cholesterol-C; MI: Myocardial Infarction PCI: Percutaneous Coronary Intervention; PTCA: Percutaneous Transluminal Coronary Angioplasty; HRQoL: Health-related Quality of Life; RCT: Randomized Clinical Trial; Tbsp: table spoon.

* NB: questions have been raised about the possibility of fabricated data; Karam et al., BMJ 2023 conducted sensitivity analysis with and without this RCT, showing similar pooled results.

Note 1: For those studies that did not report whether MI or stroke is fatal or non-fatal (Munoz 2007; Tuttle 2008), if they reported mortality, we categorized MI and stroke as non-fatal.

Note 2: When we extracted exact words from studies, we placed them in quotation marks.

**Supplementary Table 4. Characteristics of RCTs on Mediterranean dietary programs specific the co-interventions**

| **Study** | **Physical activity** | **Pharmacological management** | **Smoking cessation** | **Psychological therapy (e.g. stress management)** | **Results related to baseline pharmacological agents and co-intervention(s)** |
| --- | --- | --- | --- | --- | --- |
| **Mediterranean countries** | | | | | |
| de Lorgeril 1994; France (8) | NIR | Baseline medication use: anticoagulants, antiplatelets, beta-blockers, calcium channel blockers and ACE inhibitors. | NIR | NIR | No difference in drug therapy at baseline.  Baseline medication percentage at intervention (n=302) and control (n=303) groups respectively:  Anticoagulants (29.4% vs 26.4%), antiplatelets (62.6% vs 64.8%), beta-blockers (60.2% vs 63.4%), Calcium channel blockers (20.4% vs 21.7%) and ACE inhibitors (9.30% vs 6.10%). |
| Munoz 2007; Spain (9) | “Provide patients in the intervention group with recommendations on physical exercise (GPs offered various options, such as  walking and cycling).”  NIR in the control group. | Medications used at baseline: antiplatelets, beta-blockers, ACE inhibitors and lipid-lowering drugs.  “GPs also were asked to adjust life-saving treatments (i.e., angiotensin-converting enzyme inhibitors, aspirin, beta blockers and statins) to control risk factors according to the clinical practice guidelines, use the  drugs recommended for secondary prevention if no contraindications.”  NIR in the control group. | “Counselling to quit smoking if applicable” in the intervention group.  NIR in the control group. | NIR | Baseline medication percentage at intervention (n=515) and control (n=468) groups respectively: antiplatelets (74.5% vs 79.0%), beta-blockers (31.6% vs 38.8%), ACE inhibitors (23.0% vs 29.6%) and lipid-lowering drugs (52.9% vs 54.8%).  Control group received significantly more beta-blockers and ACE inhibitors than the intervention group.  “The hazard ratio of cardiovascular mortality was significantly higher in patients receiving ACE inhibitors.”  “A significant increase was observed in the use of all lifesaving drugs in the intervention and control groups, but increase differences between groups were not significant. The greatest variation was observed in the percentage of lipid-lowering drugs, with higher usage in the intervention group (Table 6). After adjusting for preventive drug therapy use at baseline, no significant differences between intervention and control groups were found (Table 5).” |
| Gianuzzi 2008;  Italy (10) | 30 minutes  of supervised aerobic exercise in comprehensive cardiac rehabilitation sessions with one to-  one support were held monthly from month 1 to month 6, then every 6 months for 3 years in the intervention group.  “To improve adherence  to lifestyle modification and help patients adopt a positive  role in the care of their own health, a booklet explaining how to  deal with exercise  was distributed” to the intervention group.  The targets of the intervention strategy  were to increase physical activity up to at least 3 h/wk at 60% to 75% of  the mean maximum heart rate in the intervention group.  NIR in the control group. | Pharmacological treatments were based  on current guidelines.  “Pharmacological treatment including antiplatelet agents,  ACE inhibitors or ARBs, statins, and omega-3 polyunsaturated  fatty acids, were positively recommended to all patients.” | “The targets of the intervention strategy  were to give up smoking in the intervention group, with a booklet explaining how to deal with smoking cessation  was distributed to the intervention group.”  NIR in the control group. | A booklet explaining how to deal with stress management  was distributed to the intervention group.  NIR in the control group. | Baseline medication percentage at intervention (n=1620) and control (n=1621) groups respectively: aspirin (84.1% vs 83.8%), other antiplatelets (20.2% vs 21.7%), calcium channel blockers (14.1% vs 12.0%), beta-blockers (77.5% vs 74.8%), nitrates (31.5% vs 33.6%), ACE inhibitors or ARB (60.6% vs 61.3%), diuretics (14.4% vs 16.2%), lipid-lowering drugs (70.3% vs 69.1%), oral antidiabetic drugs (7.4% vs 7.3%), and insulin (3.3% vs 3.0%).  There was no baseline difference in medication between groups.  “Most of the patients were prescribed aspirin (84%),beta blockers  (76%), statins (70%), or ACE inhibitors and ARBs (61%).”  “Lifestyle habits were similar in the 2 groups at baseline and improved in both groups during the study. The improvement was, however, significantly larger (P<0.01) in the intervention group for the scores for physical activity, stress, and dietary habits.”  “At baseline, the mean (SD) scores for physical activity were 6.7 (2.5) and 6.6 (2.4) in the intervention and usual care groups, respectively. At 6 months, it increased by 24.3% (mean score, 7.5 [2.2]) in the intervention group and by 18.2% (7.1 [2.3]) in the usual care group (Figure 3). Therefore, the 6-month score for physical activity was 6.1% higher in the intervention group (P<0.01). The difference in the level of physical activity from baseline between the 2 groups was maintained throughout the study (23.8% vs 18.8%; difference, 5%; P=0.01).”  “Compared with baseline values, the percentage of subjects  with better self/stress management (score<14.0) almost doubled at 6 months in both groups (48.1% vs 40.9%). At baseline, the mean (SD) scores for self/stress management were 16.3 (3.8) and 16.2 (3.8) in the intervention and usual care groups, respectively. At 6 months, this figure decreased by 12.1% (14.0 [3.4]) in the intervention group and 8.3% (14.5 [3.6]) in the usual care group. Therefore, the 6-month score for self/stress management adjusted for baseline was 3.8% lower in the intervention group (P<0.001). The difference in scores from baseline between the 2 groups was maintained throughout the study (14.1% vs 9.6%; difference, 4.5%; P<0.001). Similarly, the rate of patients with better self/stress management (score<14.0) was higher in the intervention group (P<0.001) (Figure 3).”  “At 6 months, smoking was significantly more likely to have been discontinued in the intensive group than in the usual care group (80.2% vs 75.1%; P=0.02). When we compared the percentage of quitters over the whole course of the study, the efficacy of the intervention against smoking declined, and eventually the absolute difference between the 2 groups was “only” 4.2% (P=0.60).”  “The use of medications at baseline was similar in the 2 groups. During follow-up, a progressive decline in the rate of use of aspirin, beta-blockers, and ACE inhibitors was seen in both groups (Figure 6). Such reduced prescription was more apparent in the usual care group. At the end of the study, the prescription of ACE inhibitors was significantly higher (P=0.02) in the intervention group than in the usual care group. The prescription  of statins increased notably during the study, and at the end it was significantly higher (P<0.001) in the intervention group (84.2%) than in the usual care group (79.1%).”  Under the methods section in the ‘analysis’: “The difference of continuous variables between intervention and control groups during and at the end of the study was adjusted for the values at baseline.” |
| Lapetra 2017; Spain (12) | NIR | NIR | NIR | NIR | NIR |
| Marcos-Forniol 2018;  Spain (13) | Physician advised low-impact aerobic activity to the intervention group.  The outcome in the intervention group was “Physical activity at least 30 minutes per day of moderate physical activity ≥3 days per week (≥6 MET’s h/wk).”  NIR in the control group. | Baseline medication use: Antiplatelets, anticoagulants, ACE inhibitors, ARBs, beta-blockers and statins. | Intervention groups received recommendation for smoking cessation.  NIR in the control group. | Intervention groups received counselling regarding stress management, sensory problems (sight and hearing), geriatric syndromes and social resources and providing and monitoring drug therapy (antidepressants, anxiolytics and/or hypnotics).  NIR in the control group. | Baseline medication percentage in intervention (n=54) and control (n=52) groups respectively: Antiplatelets or anticoagulants (100% in both groups), ACE inhibitors or ARBs (53.7 vs 53.8), beta-blockers (66.7% vs 67.3%) and statins (87.0% vs 84.6).  There was no baseline difference in medication between groups.  “No differences were observed between groups in the  use of antiplatelet agents, beta blockers and ACE inhibitors either at baseline or at the end of follow-up, whereas a significant increase in the use of lipid-lowering drugs was seen in the intervention group. At the end of follow-up, 100% of the intervention group and 82.7% of the control group were being treated with lipid-lowering drugs (RR 1.21, 95% CI 1.07–1.37). |
| PREDIMED 2018; Spain (14) | “No physical activity was advised.” | Medication use at baseline: ACE inhibitors, diuretics, other antihypertensive agents, statins, other lipid-lowering agents, Insulin, oral hypoglycemic agents, antiplatelet therapy and hormone-replacement therapy. | “Our previous experience with diabetic patients using  this approach for a behavioral intervention to quit smoking in Primary Care has been  successful.” | NIR | Baseline medication percentage in olive oil intervention (n=2543), nuts intervention (n=2454) and control (n=2450) groups respectively: ACE inhibitors (48.6%, 49.8%, 49.6%), diuretics (21.0%, 19.4%, 22.9%), other antihypertensive agents (28.5%, 28.9% 30.9%), statins (40.9%, 39.3%, 40.1%), other lipid-lowering agents (4.8%, 5.9%, 5.1%), Insulin (4.9%, 5.1%, 5.5%), oral hypoglycemic agents (30.2%, 27.7%, 30.9%), antiplatelet therapy (18.7% 20.0% 20.9%) and hormone-replacement therapy (2.8%, 2.6%, 2.7%).  “We found little difference in changes in physical activity among the three groups.”  “Drug-treatment regimens at baseline were similar for participants in the three groups and they continued to be balanced during the follow-up period (Table S6 in the Supplementary Appendix).” |
| **Non-Mediterranean countries** | | | | | |
| Singh 2002^*^; India (15) | “Patients from both groups were advised to walk briskly for a minimum of 3–4 km, or to jog intermittently for  a minimum of 10–15 minutes per day.”  “All participants with CAD were instructed to  cease exercising in the event of chest pain, substantial  sweating, weakness, or dyspnea.” | Medication use at baseline: aspirin, nitrate, verapamil, lovastatin, furosemide, disopyramide and fibrate.  “All participants with  CAD were instructed to take sublingual nitroglycerine.”  “Appropriate drugs for  angina pectoris, arrhythmias, raised blood pressure,  diabetes, and other complications were provided to both  groups.” | “Smoking and alcohol  consumption were discouraged” in all patients. | “Encouraged mental relaxation through yoga meditation techniques and  breathing exercises in both groups” in all patients. | Baseline medication percentage at intervention (n=499) and control (n=501) groups respectively: aspirin (54.0% vs 55.0%), nitrate (41.0% vs 38.0%), verapamil (15.0% vs 16.0%), lovastatin (7.0% vs 6.0%), furosemide (6.0% in both groups), disopyramide (6.0% in both groups) and fibrate (4.0% in both groups).  “In both groups, roughly half the patients were smokers at  entry to the study. Frequencies of therapeutic drug use  were closely similar in both groups. The proportions of  patients with a sedentary lifestyle were almost identical at  baseline in both groups (450 [90%] vs 462 [90%]) for  intervention and control groups, respectively. All patients  with diabetes mellitus received either glybenclamide or  glipizide.”  “At 2 years of follow-up, the intervention group, compared  with control group showed a significant reduction in the  proportion of patients taking nitrates (101 [21%] vs 152  [31%] p=0·0001), verapamil (37 [8%] vs 68 [14%],  p=0·001), and disopyramide (8 [2%] vs 29 [6%],  p<0·0001). At the end of the trial, participants in both  groups had become less sedentary, and scores for mental  relaxation showed no significant difference between groups.” |
| Søndergaard 2003; Denmark (16) | NIR | Treatment with fluvastatin (40 mg every evening) was instituted for all patients. | NIR | NIR | Baseline medication percentage at intervention (n=68) and control (n=63) groups respectively: beta blockers (56.0% vs 68.0%), calcium antagonists (22.0% vs 17.0%), ACE inhibitors (15.0% vs 22.0%), nitrate (6.0% vs 19.0%).  “During the study, 21.7% of the patients stopped smoking, with no difference in tobacco abstinence between groups after 12 months of follow-up. The use of β-blockers was significantly higher in the control group (39.7% vs 63.5%, P = 0.02), whereas there was no difference in the use of long-acting nitrates, calcium antagonists, vitamins, and ACE inhibitors at 12 months of follow-up. Both groups were >80% compliant with statin treatment.” |
| Tuttle 2008; USA (17) | In both groups “exercise was encouraged but was not a specific intervention target.” | Discharge medicine use: Aspirin, statins, ACE inhibitors and beta blockers.  “Patients with drug treatment for heart failure grade III or IV, ventricular arrhythmias, defibrillator, or uncontrolled hypertension were excluded.” | In both groups “smoking cessation was encouraged but was not a specific intervention target.” | NIR | Discharge medication percentage at intervention (n=51) and control (n=101) groups respectively: aspirin (96.0% vs 92.0%), statins (82.0% vs 85.0%), ACE inhibitors (69.0% vs 54.0%) and beta blockers (86.0% vs 71.0%).  “The use of statins, beta blockers and angiotensin-converting enzyme inhibitors or angiotensin receptor blockers did not differ between these groups at entry… or over time. Aspirin use increased from 86% (87 of 101) to 96% (68 of 71) between study entry and 24 months in dietary intervention participants (p=0.005; p=0.57 for group-by-time interaction). Medicine use and risk factors (Tables 2 and 4) at entry… varied modestly from hospital discharge because of an interval of up to 6 weeks between MI and enrollment. In usual-care controls, discharge medicines were similar to those in dietary intervention patients except that beta blocker use was lower. During the follow-up period, more percutaneous coronary interventions and coronary artery bypass operations were performed in usual-care controls (n= 24 and 7 respectively) than in dietary intervention patients (n=15 and 4 respectively). The total number of coronary revascularization procedures was similar between the Mediterranean-style (n=11) and low-fat (n=8) diet groups.”  “Unadjusted odds ratio for the primary outcome was 0.33 (95% CI 0.18 to 0.60, p=0.001) for dietary intervention compared with usual care. The adjusted odds ratio was 0.28 (95% CI 0.13 to 0.63, p=0.002) after controlling for baseline frequencies of obesity, smoking, and cardiac rehabilitation enrollment; low-density lipoprotein cholesterol level; and discharge medicine use.” |
| Lehmann 2011; Germany (18) | “Regular exercise and increased daily activity were strongly recommended” in the intervention group. | Baseline medication use: aspirin, statins,  beta-blockers and  ACE inhibitors.  “According to current guidelines all patients were recommended to continue their medication with aspirin and analogues, statins, ACE inhibitor /AT-1-blockers and beta-blockers.” | NIR | “Patients in the advice group received written information about stress management and dietary advice by means of a booklet that was mailed shortly after randomization.”  “Patients in intervention group practiced various relaxation techniques according to personal choice. Techniques taught included mindfulness meditation, guided imagery, yoga breathing techniques and body scan. Further elements included CBT (cognitive restructuring) and psychoeducational approaches (coping skills training).” | Baseline medication percentage at intervention (n=47) and control (n=49) groups respectively: aspirin (97.9% vs 98.1%), statins (85.1% vs 87.6%), beta-blockers (76.6% vs 77.6%) and ACE inhibitors (50.0% vs 49.1%).  “Symptomatic improvement of the lifestyle modification and stress reduction intervention group (LG) was reflected by decreased intensity of anti-ischemic medication after three years.”  “While the proportions of medically treated patients did not change, there were significantly more frequent dose-reductions or discontinuations of anti-ischemic medications, especially nitrates (LG vs. advice group (AG):18 of 21 vs. 6 of 18, p=0.0009) and calcium-antagonists (10 of 13 vs. 2 of 12, p=0.003). The prescribed dose of statins remained almost unchanged in both groups. ACE-inhibitors were prescribed to 25 patients in LG and reduced or discontinued in 10 patients, while they were reduced in 6 of 23 patients in AG, p=0.31. Beta-blockers were reduced or discontinued in 16 of 37 vs.10 of 37 patients, p=0.15.” |
| Matz 2015; Austria (19) | “In the intervention group the goal is to perform moderate or vigorous physical activity at least three to five times per week. Participants of the intervention group participate in regular group meetings (six meetings during the first year and two meetings during the second year), which include general counseling, detailed instructions for exercises, and group activity planning. The patients are encouraged to participate in local activity groups in the participant's communities. Existing possibilities in the local communities are presented and individually recommended to the participants.”  One of the study goals was performing moderate or vigorous physical activity at least 3× to 5×/week.  NIR in the control group. | In all patients “compliance with pharmacological  treatment including lipid-lowering drugs, antithrombotic/oral  anticoagulation therapy, and glucose-lowering drugs was targeted according  to the treatment goals of stroke prevention European Stroke  Organization (ESO) guideline.” | The patients who smoked were encouraged to stop smoking, and smoking cessation counseling was offered in the intervention group.  NIR in the control group. | NIR | Based on the initial publication reporting study baseline characteristics only for this trial, there was no significant between group differences in the distribution of current or former smokers of for physical activity (21). There was no information regarding baseline medications. |
| Singh 2017*; India  (20) | All patients were advised to participate regularly in physical activity.  “Among patients in group A, however, recommendations were reinforced daily on what to do while in the hospital and also weekly after discharge by a dietician.”  “Patients were counselled regularly at separate counseling sessions and asked to complete a questionnaire about dietary intake, smoking, alcohol intake, and drug intake to enforce drug and dietary compliance and to stop or reduce smoking and alcohol intake in both groups.” | Drug treatments, including propranolol, verapamil, nitrates, furosemide, aspirin and antidiabetic medications (mainly metformin). | All patients were advised to stop smoking and reduce alcohol intake.  Otherwise, see “Physical activity” column for additional details | All patients were advised to practice yoga to relieve mental stress.  Otherwise, see “Physical activity” column for additional details | At baseline “There were slightly more smokers (74, 36% vs. 70, 35%) and fewer alcohol consumers (45, 22.0%, vs. 50, 24.7%) in group A, compared to group B.”  “Drug treatments, including propranolol (daily dose; N, proportion of subjects – Group A vs. B) (40-240 mg; 95, 46%, vs. 88, 44%), verapamil (20-60 mg; 50, 25%, vs. 55, 27%), nitrates (60-180 mg; 200, 98%, vs. 196, 97%), furosemide (20-80 mg; 29, 14%, vs. 36, 18%), and aspirin (175-355 mg; 29, 14%, vs. 197, 18%), as well as antidiabetic medications, mainly metformin (0.50-2.0 g/day) did no differ (p>0.05) between the two groups.” |

Abbreviations: ACE: Angiotensin Converting Enzyme; ARB: Angiotensin-II Receptor Blockers*,* GP: general Practitioner; NIR: No Information Reported.

* Questions have been raised about the possibility of fabricated data in Singh et al; Karam et al., BMJ 2023 conducted sensitivity analysis with and without this RCT, showing similar pooled results

**References**

1. Doundoulakis I, Farmakis IT, Theodoridis X, Konstantelos A, Christoglou M, Kotzakioulafi E, et al. Effects of dietary interventions on cardiovascular outcomes: a network meta-analysis. Nutrition Reviews. 2024;82(6):715-25.

2. Karam G, Agarwal A, Sadeghirad B, Jalink M, Hitchcock CL, Ge L, et al. Comparison of seven popular structured dietary programmes and risk of mortality and major cardiovascular events in patients at increased cardiovascular risk: systematic review and network meta-analysis. bmj. 2023;380.

3. Wallerer S, Papakonstantinou T, Morze J, Stadelmaier J, Kiesswetter E, Gorenflo L, et al. Association between substituting macronutrients and all-cause mortality: a network meta-analysis of prospective observational studies. EClinicalMedicine. 2024;75:102807.

4. Bonekamp N, Cruijsen E, Geleijnse J, Winkels R, Visseren F, Morris P, et al. Diet in secondary prevention: the effect of dietary patterns on cardiovascular risk factors in patients with cardiovascular disease: a systematic review and network meta-analysis. Nutrition Journal. 2024;23(1):18.

5. Lesser LI. In adults at CV risk, Mediterranean-style or low-fat dietary programs vs. minimal interventions reduce all-cause mortality. Ann Intern Med. 2023;176(7):Jc78.

6. Li W, Liu H, Wang X, Liu J, Xiao H, Wang C, et al. Interventions for reducing blood pressure in prehypertension: A meta-analysis. Front Public Health. 2023;11:1139617.

7. Bonekamp N, van Damme I, Geleijnse J, Winkels R, Visseren F, Morris P, et al. Effect of dietary patterns on cardiovascular risk factors in people with type 2 diabetes. A systematic review and network meta-analysis. Diabetes Research and Clinical Practice. 2023;195:110207.

8. De Lorgeril M, Renaud S, Salen P, Monjaud I, Mamelle N, Martin J, et al. Mediterranean alpha-linolenic acid-rich diet in secondary prevention of coronary heart disease. The lancet. 1994;343(8911):1454-9.

9. Munoz M-A, Vila J, Cabañero M, Rebato C, Subirana I, Sala J, et al. Efficacy of an intensive prevention program in coronary patients in primary care, a randomised clinical trial. International journal of cardiology. 2007;118(3):312-20.

10. Giannuzzi P, Temporelli PL, Marchioli R, Maggioni AP, Balestroni G, Ceci V, et al. Global Secondary Prevention Strategies to Limit Event Recurrence After Myocardial Infarction: Results of the GOSPEL Study, a Multicenter, Randomized Controlled Trial From the Italian Cardiac Rehabilitation Network. Archives of Internal Medicine. 2008;168(20):2194-204.

11. Knoops KT, De Groot LC, Kromhout D, Perrin A-E, Moreiras-Varela O, Menotti A, et al. Mediterranean diet, lifestyle factors, and 10-year mortality in elderly European men and women: the HALE project. Jama. 2004;292(12):1433-9.

12. Lapetra J, Lozano-Rodriguez J, Miro-Moriano L, Ortega-Calvo M, Santos-Lozano J, Garcia-Corte F. Effect of a Mediterranean diet on the primary prevention of atrial fibrillation and major cardiovascular events in hypertensive patients with high cardiovascular risk: results of ICFAMED randomized trial. European Journal of Clinical Investigation. 2018;48:182-3.

13. Marcos-Forniol E, Meco JF, Corbella E, Formiga F, Pintó X. Secondary prevention programme of ischaemic heart disease in the elderly: a randomised clinical trial. European Journal of Preventive Cardiology. 2018;25(3):278-86.

14. Estruch R, Ros E, Salas-Salvadó J, Covas M-I, Corella D, Arós F, et al. Primary prevention of cardiovascular disease with a Mediterranean diet supplemented with extra-virgin olive oil or nuts. New England journal of medicine. 2018;378(25):e34.

15. Singh RB, Dubnov G, Niaz MA, Ghosh S, Singh R, Rastogi SS, et al. Effect of an Indo-Mediterranean diet on progression of coronary artery disease in high risk patients (Indo-Mediterranean Diet Heart Study): a randomised single-blind trial. The Lancet. 2002;360(9344):1455-61.

16. Søndergaard E, Møller JE, Egstrup K. Effect of dietary intervention and lipid-lowering treatment on brachial vasoreactivity in patients with ischemic heart disease and hypercholesterolemia. American Heart Journal. 2003;145(5):903.

17. Tuttle KR, Shuler LA, Packard DP, Milton JE, Daratha KB, Bibus DM, et al. Comparison of Low-Fat Versus Mediterranean-Style Dietary Intervention After First Myocardial Infarction (from The Heart Institute of Spokane Diet Intervention and Evaluation Trial)††Conflicts of interest: Dr. Bibus has received consulting fees from companies that make fish oil, Coromega Co., Vista, CA, and Enreco, Inc., Newton, WI. He also has equity ownership in Lipid Technologies, LLC, Austin, MN, the company that performed the plasma fatty acid analysis. Dr. Bibus owns patents related to lipid therapy. He has served as an expert witness regarding lipids and fatty acids. The American Journal of Cardiology. 2008;101(11):1523-30.

18. Lehmann N, Paul A, Moebus S, Budde T, Dobos GJ, Michalsen A. Effects of lifestyle modification on coronary artery calcium progression and prognostic factors in coronary patients—3-Year results of the randomized SAFE-LIFE trial. Atherosclerosis. 2011;219(2):630-6.

19. Matz K, Teuschl Y, Firlinger B, Dachenhausen A, Keindl M, Seyfang L, et al. Multidomain lifestyle interventions for the prevention of cognitive decline after ischemic stroke: randomized trial. Stroke. 2015;46(10):2874-80.

20. Singh RB, Saboo B, Mahashwari A, Bharatdwaj K, Verma N, Hristova K, et al. Effects of Indo-Mediterranean Style Diet and Low Fat Diet on Incidence of Diabetes in Acute Coronary Syndromes. World Heart Journal. 2017;9(1):25-36.

21. Brainin M, Matz K, Nemec M, Teuschl Y, Dachenhausen A, Asenbaum-Nan S, et al. Prevention of poststroke cognitive decline: ASPIS–a multicenter, randomized, observer-blind, parallel group clinical trial to evaluate multiple lifestyle interventions–study design and baseline characteristics. International Journal of Stroke. 2015;10(4):627-35.
